# Towards the automation of *in situ* experimental phasing

**DOI:** 10.1107/S205979832001178X

**Published:** 2020-09-01

**Authors:** Dorothee Liebschner

**Affiliations:** a Molecular Biosciences and Integrated Bioimaging, Lawrence Berkeley National Laboratory, Berkeley, California 94720-8235, USA

**Keywords:** commentary, experimental phasing, high-throughput, SAD, SIRAS

## Abstract

A new approach to *in situ* experimental phasing introduced by Lawrence *et al.* (2020, *Acta Cryst.* D**76**, 790–801) will be helpful for the macromolecular crystallography community.

The predicament of a diffraction experiment lies in the loss of phase information. Fortunately, it is possible to recover phases, either experimentally or through computations, by assuming some prior knowledge about the molecular structure (*i.e.* the electron density). Three methods exist to recover phases from a diffraction experiment: (1) direct methods (Karle & Hauptman, 1956 ▸), which rely on phase relationships that can be formulated by assuming the positivity and atomicity of the electron density. (2) Molecular replacement (MR) (Rossmann, 1972 ▸), which is based on the observation that a previously known structure can provide initial phase estimates for the new structure. (3) Experimental phasing (Fig. 1 ▸), which exploits the properties of a few special atoms in the macromolecule, such as anomalous scattering, a large number of electrons, or a combination of both (reviewed by Dauter & Dauter, 2017 ▸). Direct methods can be only applied if the resolution of the diffraction data is very high (better than ~1.2 Å), which only occurs in exceptional cases for macromolecular crystals. MR is widely applied, thanks to well established algorithms and protocols, and the availability of a trove of models in the Protein Data Bank (Berman *et al.*, 2000 ▸). However, it may happen that no known structures can be found that provide good enough phase estimates for the new structure. In this case, the only possibility is to resort to experimental phasing.

The basic idea of experimental phasing is to locate the position of a subset of atoms (‘marker atoms’) that have particular properties. The marker atoms can be purposely incorporated or natively present in the sample. If the marker atoms have many electrons, such as heavy-metal salts or metal clusters, isomorphous replacement tries to detect the difference in amplitudes for the native and the derivative crystal. The approach can be done with one (SIR) or several derivatives (MIR). The intensity differences can reach as much as 15–25% (Crick & Magdoff, 1956 ▸), but incorporating the marker atoms may also cause non-isomorphism. This can result in similar changes of intensity, therefore complicating the phasing process. Other challenges of SIR/MIR are the toxicity of some heavy atoms, finding the appropriate marker atom type, and the deterioration of the crystalline order when the heavy atoms bind to the macromolecule.

Another experimental phasing approach exploits the difference in intensities between reflections related by Friedel’s law for anomalous scatterers, such as seleno­methio­nine. The approach can be done at one (SAD) or several (MAD) energies near the absorption edge of the marker atom. As a complete SAD/MAD dataset can be collected from a single crystal, isomorphism is less of a problem than for SIR/MIR. However, the anomalous differences in intensity are much smaller, so the experimental data have to be measured very accurately.

The single and multiple isomorphous replacement with anomalous scattering methods (SIRAS and MIRAS) combine the heavy-atom and anomalous scatterer approaches by also using the dispersive signal of the heavy atom.

While the above-described methods are well established, they still face technical and computational challenges. Therefore, a significant amount of effort is being directed to achieve advances in experimental phasing. For example, SAD phasing is now increasingly used with sulfur and ions that can exist natively in macromolecules (Rose *et al.*, 2015 ▸). Furthermore, it was shown that combining data sets from multiple crystals can improve the chances of determining the substructure (Liu *et al.*, 2011 ▸). Some synchrotron beamlines are specifically designed to collect data near the absorption edge of sulfur (Wagner *et al.*, 2016 ▸), or to collect accurate and high-multiplicity data (Olieric *et al.*, 2016 ▸). A new metric assesses the potential of SAD data to successfully solve the structure (Terwilliger *et al.*, 2016*a* ▸,*b* ▸). Recently, a new approach to experimental phasing was suggested by Lawrence and colleagues in an *Acta Cryst. D* article (Lawrence *et al.*, 2020 ▸). The procedure makes use of robotics to automatically soak hundreds of samples in heavy-atom solutions, producing isomorphous derivative crystals. Partial diffraction data are collected *in situ* at room temperature (RT) and combined to maximize the anomalous signal.

Lawrence *et al.* proceeded as follows to demonstrate their approach. Crystals of tetragonal lysozyme and proteinase K were prepared in 96-well sitting-drop plates with a crystallization robot. Some wells containing crystals were automatically soaked with halide (NaBr, KBr, NaI, KI) and heavy-metal solutions [K_2_Pt(NO_2_)_4_, KAuCl_4_·H_2_O, K_2_IrCl_6_, SmCl_3_0·6H_2_O] by using a liquid handler. A subset of native and derivative crystals was manually cryo-cooled for control measurements at 100 K. To perform *in situ* data collection at RT, the crystallization plates were directly mounted on a goniometer and 21–116 partial data sets were collected on different crystals. Native data sets were collected around 12 keV incident X-ray energy while the energies for the derivative crystals were adapted to the type of heavy atom. Phasing was attempted with the SAD and SIRAS methods. Phasing was considered a success if three indicators were successful: anomalous signal strength (as expressed by *d*′′), substructure solution and the fraction of residues that could be built automatically.

For the 100 K control measurements, phasing of lysozyme works in all cases for both SAD and SIRAS. Proteinase K was successful in four cases for SAD and in four cases for SIRAS. At RT, lysozyme phasing is successful in half of SAD and SIRAS cases. Proteinase K only works for the gold derivative. Generally, SIRAS, which can solve the phase ambiguity for the substructure, results in a larger fraction of residues built.

Several observations can be made from the results. Isomorphism, as measured by the associated unit-cell variability, is markedly better at RT than at 100 K. As this new phasing approach relies on isomorphism, it corroborates the choice of performing the experiments at RT. Anomalous difference maps show clear peaks for at least a fraction of marker atoms. This means that the soaking protocol resulted in binding, which is a critical prerequisite for the method. Crystal size influences the phasing success, as showed by the results for the small proteinase K crystals (10 × 10 × 10 µm). Combining a large number of data sets may improve phasing outcome. The heavy-atom substrates have different propensities to bind to the macromolecule. Therefore, using a combination of substrates provides the largest chances of success that one may bind to the sample.

The high-throughput phasing method devised by Lawrence *et al.* will certainly be helpful for the macromolecular crystallography community as it reduces human intervention in the sample preparation process to a minimum, therefore increasing the chances of producing isomorphous crystals in reproducible conditions. This is advantageous for both SIRAS, which relies on isomorphism, and SAD, which benefits from it when data sets are combined from different crystals. Using two phasing methods, including one that breaks the phase ambiguity, increases the chance of phasing success. The high-throughput approach may either provide a phasing dataset or, if phasing was not achieved, it may help reveal promising conditions that can be further optimized. The authors suggest that the method may be also extended to serial crystallography. While some limitations exist, for example the *in situ* data-collection method will be challenging for low-symmetry space groups and suffers from higher background noise from the crystallization plate (thus reducing the achievable anomalous signal), the high-throughput phasing method has the potential to greatly facilitate heavy-atom phasing experiments.

## Figures and Tables

**Figure 1:**
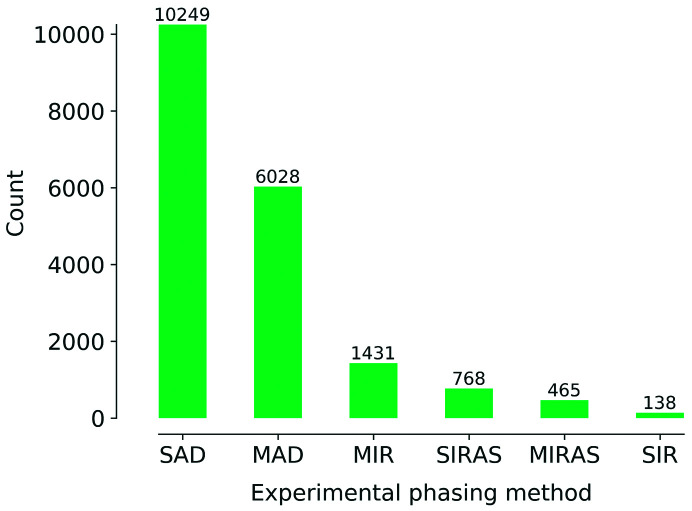
Count of experimental phasing methods for X-ray models deposited in the PDB, in descending order. The counts are obtained from the RCSB advanced search, so they depend on correct annotations in the metadata. Not shown: RIP phasing, 7 entries. Other phasing methods: MR, 104,673 entries; direct methods, 166 entries (search for keyword and resolution better than 1.4 Å).
